# Two variants of Interleukin-1B gene are associated with the decreased risk, clinical features, and better overall survival of colorectal cancer: a two-center case-control study

**DOI:** 10.18632/aging.101695

**Published:** 2018-12-19

**Authors:** Haihua Qian, Dan Zhang, Chuanqing Bao

**Affiliations:** 1 Department of Anorectal Surgery, Jiangsu Provincial Hospital of Traditional Chinese Medicine, Nanjing, Jiangsu 210029, China; 2 Department of General Surgery, Third Affiliated Hospital of Nantong University, Wuxi, China; * Equal contribution

**Keywords:** IL-1B, colorectal cancer, two-center case-control study

## Abstract

Interleukin (IL)-1B reportedly promotes the stemness and invasiveness of colon cancer cells. Several studies have investigated the association between IL-1B gene polymorphisms and colorectal cancer (CRC) risk, but report conflicting findings. Here, this association was explored in a hospital-based case-control study involving 527 CRC cases and 639 controls from two Chinese Han populations. Genotyping was done by matrix-assisted laser desorption/ionization time-of-flight mass spectrometry. The IL-1B expression in CRC patients and controls were obtained from the GEPIA database and the mRNA expressions of different genotypes were downloaded from the GTEx portal database. The relationship of two IL-1B gene loci with clinical parameters and overall survival were analyzed using the Chi-square test and Kaplan-Meier analysis with the log-rank test respectively. It was found that the IL-1B mRNA levels in CRC patients were significantly higher than in controls. Bioinformatic analysis suggested that rs1143634 and rs1143623 polymorphisms decreased the IL-1B mRNA expression. The two polymorphisms were associated with decreased risk for CRC. Stratified analyses revealed the IL-1B gene rs1143623 and rs1143634 polymorphisms decreased the risk of CRC among females, smokers and drinkers. Moreover, the CC and/or GC genotype of rs1143623 polymorphism were correlated with decreased risk among CRC patients with tumor size ≥5cm, TNM stage III+IV, and rectal cancer. For rs1143634 polymorphism, the CT genotype reduced the risk of colorectal adenocarcinoma. The CRC patients carrying CC genotype of rs1143623 polymorphism were associated with better overall survival. In conclusion, IL-1B gene rs1143623 and rs1143634 polymorphisms are associated with decreased risk for CRC patients and thereby play important roles in the etiology of CRC.

## Introduction

Colorectal cancer (CRC) ranks third in incidence and second in mortality among all cancers [1]. Over 1.8 million new CRC patients and 881,000 deaths were estimated to occur in 2018 [1]. The incidence and mortality of CRC in China are increasing in the recent decade. However, the etiology of CRC is still poorly understood but may be attributed to dietary patterns, smoking, lifestyle factors, drinking and other factors [2,3]. Moreover, the elevated expression of proinflammatory cytokines is associated with the tumorigenesis of sporadic CRC [4].

Chronic inflammation is one of the hallmarks of CRC, which arises following prolonged inflammation [5,6]. Any substantial shift of the bacterial population could considerably affect inflammatory responses and contribute to CRC development [7]. Interleukin (IL)-1 is a group of potent proinflammatory cytokines including IL-1B which is activated by caspase-1 upon inflammasome activation in tumor cells [8]. IL-1B induces a host of growth factors and angiogenic factors and promote tumor growth and metastasis [8]. A mouse CRC model shows IL-1B arising from tumor-infiltrating neutrophils plays an pivotal role in tumorigenesis [9].

Single-nucleotide polymorphisms (SNPs) in the promoter regions of cytokine genes may affect the protein IL-1 expressions, thereby influencing the risk of cancer. Up to date, several studies have investigated the association between IL-1B gene polymorphisms and CRC risk, but report conflicting findings. In this study, we selected two SNPs (rs1143634, rs1143623 polymorphisms). The rs1143623 polymorphism is located at the transcription factor binding site of the IL-1B gene, and nucleotide changes (G to C) may affect the transcription factor binding. The rs1143634 polymorphism causes synonymous mutation when the nucleotide changes from C to T. A Danish study [10] firstly found the IL-1B gene rs1143623 polymorphism increased the risk of CRC and a Russian study [11] suggested was not associated with CRC risk. Regarding rs1143634 polymorphism, an American study and a Romanian study found no association between this SNP and CRC risk [12,13], while a Chinese study [14] showed that it was related to increased risk of CRC. To validate the inconsistences among these studies, we designed this two-center case-control study to clarify the potential association between two IL-1B gene variants (rs1143634, rs1143623 polymorphisms) and CRC risk. In addition, we explored the links of the IL-1B gene variants with clinical features and overall survival (OS) of CRC.

## RESULTS

### Characteristics of the study population

Baseline characteristics of the study population and their statistical significance were summarized in Table 1. The CRC patients and healthy controls were aged 56.46±11.70 and 56.00±10.59 years, respectively. The male-female ratios in the cases and controls were 1.45:1 and 1.39:1 respectively. No significant between-group difference was found in smoking, drinking or family history of cancers. Most CRC patients were rectal carcinoma or adenocarcinoma and in the TNM stage II and III. Other clinical parameters (tumor size, CEA, CA199 and differentiation) were also recorded.

**Table 1:** Patient demographics and risk factors in colorectal cancer.

Characteristics	Case (N=527)	Control (N=639)	*P*
Age	56.46±11.70	56.00±10.59	0.490
Gender			0.733
Female	215(40.8%)	267(41.8%)	
Male	312(59.2%)	372(58.2%)	
Smoking			0.371
Yes	286(54.3%)	330(51.6%)	
No	241(45.7%)	309(48.4%)	
Drinking			0.156
Yes	308(58.4%)	347(54.3%)	
No	219(41.6%)	292(45.7%)	
Family history of cancer			0.106
Yes	65(12.3%)	100(15.6%)	
No	462(88.4%)	539(84.7%)	
			
Tumor size			
<5cm	306(58.1%)	—	
≥5cm	221(41.9%)	—	
CEA			
Positive	143(27.1%)	—	
Negative	384(72.9%)	—	
CA199			
Positive	152(28.8%)	—	
Negative	375(71.2%)	—	
Tumor location			
Rectum	303(57.5%)	—	
Colon	224(42.5%)	—	
Differentiation			
Well	244(58.3%)	—	
Moderate	204(41.7%)	—	
Poor	79(59.3%)	—	
Adenocarcinoma			
Yes	301(57.1%)	—	
No	226(42.9%)	—	
TNM classification			
I	65(16.9%)	—	
II	197(34.0%)	—	
III	162(41.0%)	—	
IV	103(8.1%)	—	

CEA: carcino-embryonic antigen; CA199: carbohydrate antigen 19-9; TNM: tumor node metastasis.

### Bioinformatic analysis

**Figure 1:**
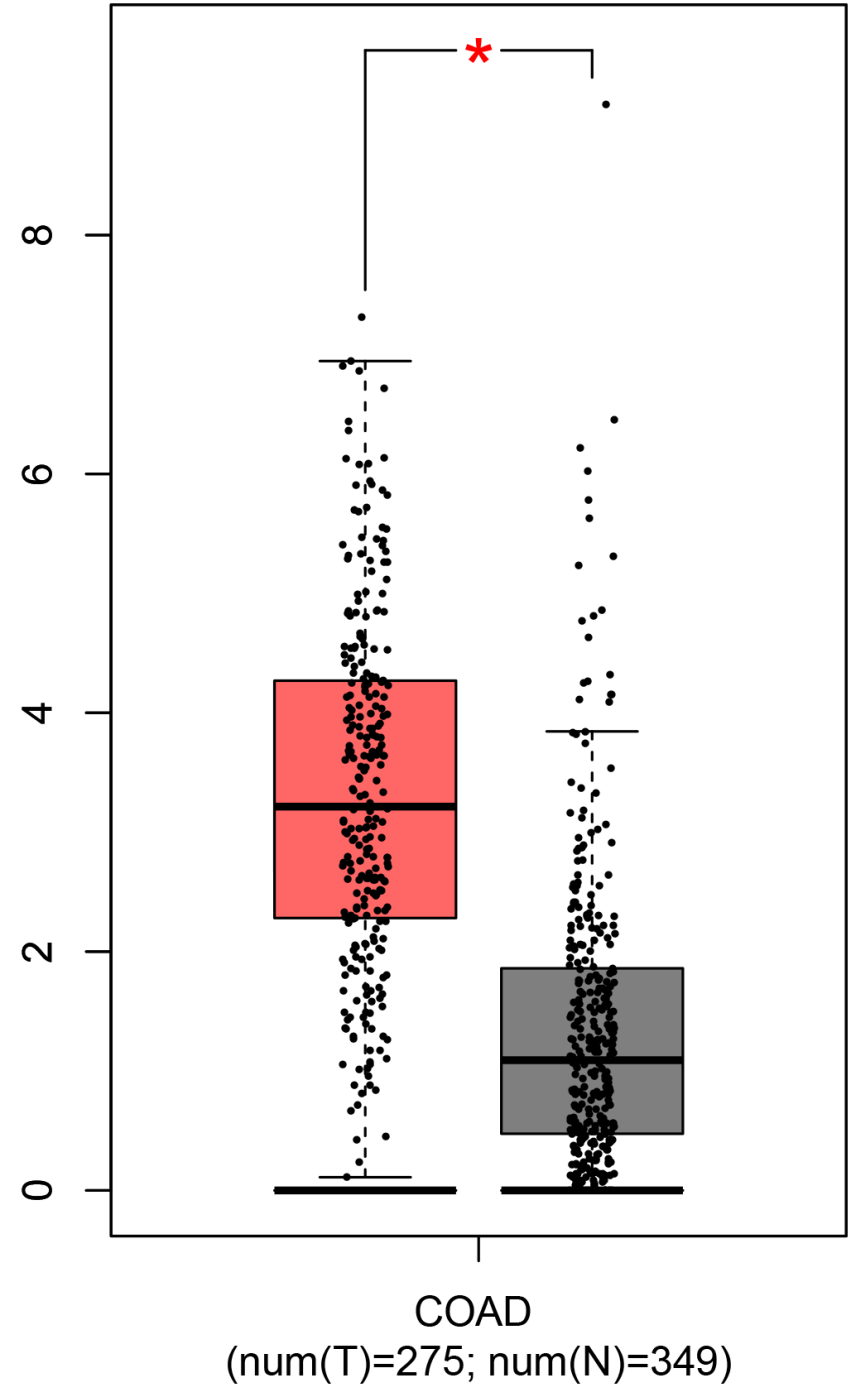
**TCGA data confirmed the IL-1B expressions in rectal cancer patients were significantly higher than in the control, which was analyzed by GEPIA.** T: tumor samples; N: paired normal tissues.

**Figure 2:**
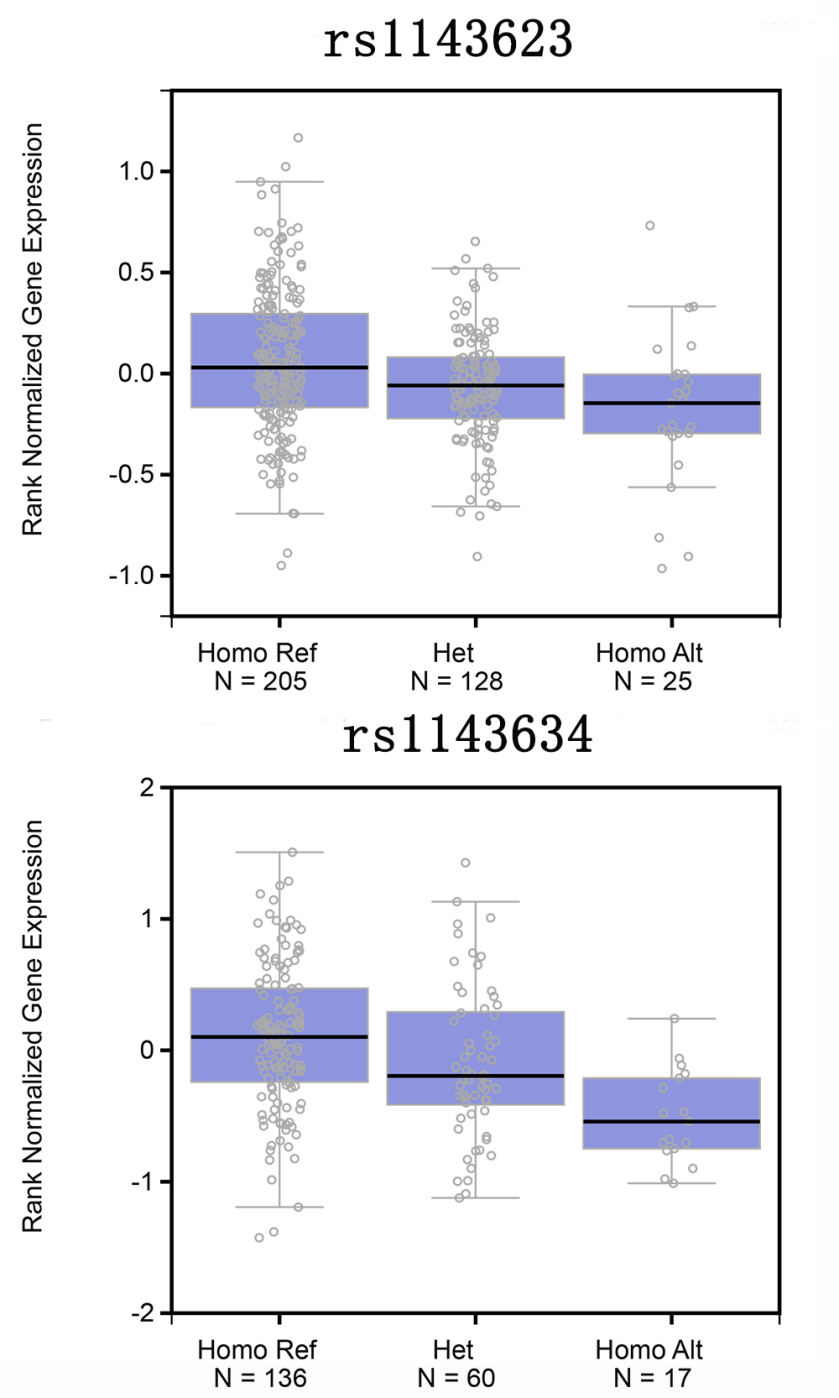
**A association between IL-1B mRNA expression levels and genotypes of rs1143623 or rs1143634 polymorphisms.** Data about genotypes and mRNA expression in esophagus-mucosa were obtained from the public GTEx portal database.

GEPIA database analysis based on data from The Cancer Genome Atlas (TCGA) showed the IL-1B mRNA levels in the CRC patients were significantly higher than in the controls (Figure 1). The data from the International HapMap Project and GTEx portal showed the mutant genotype of the two polymorphisms could decrease IL-1B gene expression (Figure 2). We hypothesized the IL-1B rs1143623 and rs1143634 polymorphisms conferred susceptibility to CRC by altering the IL-1B expression.

### Association of IL-1B gene polymorphisms with CRC risk and clinical parameters of CRC

Table 2 shows the genotype and allele distributions of the two polymorphisms (rs1143623, rs1143634) polymorphisms in CRC cases and controls. No significant deviation from HWE was found for the either SNP in the controls. The rs1143623 polymorphism was associated with the decreased risk of CRC in the homozygous, dominant and allelic models. Similar findings were observed for the rs1143634 polymorphism, as it decreased the risk of CRC under the five models. This association remained true after the adjustment of sex and age.

**Table 2:** Genotype frequencies of IL-1B gene polymorphisms in colorectal cancer cases and controls.

Models	Genotype	Case (n, %)	Control (n, %)	OR (95% CI)	*P*-value	Adjusted OR (95% CI)*	*P*-value
rs1143623							
Co-dominant	GG	306 (58.1%)	329 (51.5%)	1.00(reference)	-	1.00(reference)	-
Heterozygote	GC	188 (35.7%)	251 (39.3%)	0.81(0.63,1.03)	0.083	0.76(0.47,1.22)	0.256
Homozygote	CC	32 (6.1%)	57 (8.9%)	**0.60(0.38,0.96)**	**0.032**	**0.61(0.38,0.97)**	**0.036**
Dominant	GG	306 (58.1%)	329 (51.5%)	1.00(reference)	-	1.00(reference)	-
	GC+CC	220 (41.7%)	308 (48.2%)	**0.77(0.61,0.97)**	**0.026**	**0.77(0.61,0.97)**	**0.026**
Recessive	GC+GG	494 (93.7%)	580 (90.8%)	1.00(reference)	-	1.00(reference)	-
	CC	32 (6.1%)	57 (8.9%)	0.66(0.42,1.03)	0.069	0.84(0.66,1.08)	0.183
Allele	G	800 (75.9%)	909 (71.1%)	1.00(reference)	-		
	C	252 (23.9%)	365 (28.6%)	**0.78(0.65,0.95)**	**0.011**		
Rs1143634							
Co-dominant	CC	399 (75.7%)	447 (70.0%)	1.00(reference)	-	1.00(reference)	-
Heterozygote	CT	119 (22.6%)	168 (26.3%)	0.79(0.61,1.04)	0.094	0.79(0.60,1.04)	0.089
Homozygote	TT	8 (1.5%)	22 (3.4%)	**0.41(0.18,0.93)**	**0.032**	**0.41(0.18,0.94)**	**0.036**
Dominant	CC	399 (75.7%)	447 (70.0%)	1.00(reference)	-	1.00(reference)	-
	CT+TT	127 (24.1%)	190 (29.7%)	**0.75(0.58,0.97)**	0.031	**0.75(0.58,0.97)**	0.030
Recessive	CT+CC	518 (98.3%)	615 (96.2%)	1.00(reference)	-	1.00(reference)	-
	TT	8 (1.5%)	22 (3.4%)	**0.43(0.19,0.98)**	0.044	**0.44(0.19,1.00)**	0.049
Allele	C	917 (87.0%)	1062 (83.1%)	1.00(reference)	-		
	T	135 (12.8%)	212 (33.2%)	**0.74(0.58,0.93)**	**0.010**		

*Adjusted for sex and age. Bold values are statistically significant (*P* < 0.05)

Stratified analyses according to age, sex, smoking and drinking were also conducted (Table 3). The IL-1B gene rs1143623 polymorphism decreased the risk of CRC among female, but not among male. The significant findings were further confirmed among the individuals <50 years, smokers and drinkers. Subgroup analyses by age revealed the rs1143634 decreased risks in individuals ≥ 50 years but not in < 50 years. This association held true in females, smokers and drinkers.

**Table 3:** Stratified analyses between IL-1B gene polymorphisms and risk of colorectal cancer.

Variable	case/control			Homozygous model	Dominant model	Recessive model
Rs1143623	GG	GC	CC	CC vs. GG	CC+GC vs. GG	CC vs. GC+GG
Age (years)						
<50	101/94	65/74	8/18	**0.44(0.21,0.91); 0.024**	0.90(0.64,1.26); 0.530	**0.44(0.21,0.90); 0.024**
≥50	205/235	123/177	24/39	0.75(0.41,1.37); 0.351	**0.67(0.48,0.92); 0.014**	0.89(0.50,1.61); 0.709
Sex						
Female	122/152	84/90	9/25	**0.45(0.20,1.00); 0.049**	1.01(0.70,145); 0.967	**0.42(0.19,0.93); 0.032**
Male	184/177	104/161	23/32	0.69(0.39,1.23); 0.208	**0.63(0.47,0.86); 0.003**	0.84(0.48,1.47); 0.550
Smoking						
No	146/169	81/118	14/21	0.77(0.38,1.57); 0.475	0.79(0.56,1.11); 0.180	0.84(0.42,1.69); 0.631
Yes	160/160	107/133	18/36	**0.50(0.27,0.92); 0.025**	0.74(0.54,1.02); 0.064	**0.55(0.30,0.99); 0.046**
Drinking						
No	122/156	83/123	14/13	1.38(0.62,3.04); 0.428	0.91(0.64,1.30); 0.608	1.47(0.67,3.19); 0.334
Yes	184/173	105/128	18/44	**0.39(0.21,0.69); 0.001**	**0.67(0.49,0.92); 0.012**	**0.43(0.24,0.76); 0.004**
Rs1143634	CC	CT	TT	TT vs. CC	TT+CT vs. CC	TT vs. CT+CC
Age (years)						
<50	128/133	45/47	1/6	0.34(0.09,1.25); 0.104	0.88(0.60,1.27); 0.484	0.34(0.09,1.26); 0.108
≥50	271/314	74/121	7/16	0.46(0.16,1.34); 0.155	**0.64(0.44,0.93); 0.018**	0.51(0.18,1.47); 0.212
Sex						
Female	155/185	55/66	4/15	**0.32(0.10,0.98); 0.046**	0.87(0.58,1.29); 0.490	**0.32(0.10,0.98); 0.045**
Male	244/262	64/102	4/7	0.61(0.18,2.12); 0.440	**0.67(0.47,0.95); 0.025**	0.68(0.20,2.33); 0.534
Smoking						
No	181/225	56/74	4/9	0.55(0.17,1.82); 0.330	0.90(0.61,1.32); 0.587	0.56(0.17,1.84); 0.341
Yes	218/222	63/94	4/13	**0.31(0.10,0.98); 0.045**	**0.64(0.45,0.91); 0.014**	**0.35(0.11,1.07); 0.066**
Drinking						
No	170/206	45/79	3/7	0.52(0.13,2.04); 0.348	0.68(0.45,1.02); 0.060	0.57(0.15,2.22); 0.417
Yes	229/241	74/89	5/15	**0.35(0.13,0.98); 0.046**	0.80(0.57,1.13); 0.202	**0.36(0.13,1.01); 0.053**

Bold values are statistically significant (*P* < 0.05)

We explored the association between IL-1B gene polymorphisms and clinical characteristics (Table 4). The CC and/or GC genotype of rs1143623 polymorphism was correlated with decreased risk for patients with tumor size ≥ 5cm, TNM stage III+IV, and rectal cancer. Moreover, the CT genotype of rs1143634 polymorphism reduced the risk of colorectal adenocarcinoma compared to other subtypes of CRC. Similar result was found in the CT+TT genotype.

**Table 4:** The associations between IL-1B gene polymorphisms and clinical characteristics of colorectal cancer.

Characteristics	Genotype distributions			
**rs1143623** Tumor size	GG	GC	CC	GC+CC
≥5cm /<5cm	139/167	73/115	8/24	81/139
OR (95%CI); *P*-value	1.0 (reference)	0.83(0.57,1.20); 0.310	**0.40(0.17,0.92); 0.027**	**0.70(0.49,1.00); 0.048**
CEA				
Positive/ Negative	86/220	45/143	11/21	56/164
OR (95%CI); *P*-value	1.0 (reference)	0.86(0.57,1.31); 0.480	1.34(0.62,2.90); 0.456	0.87(0.59,1.29); 0.499
CA199				
Positive/ Negative	85/221	55/133	12/20	67/153
OR (95%CI); *P*-value	1.0 (reference)	1.08(0.72,1.61); 0.723	1.56(0.73,3.33); 0.247	1.14(0.78,1.67); 0.504
Differentiation				
Poor/Well+moderate	43/263	32/156	4/28	36/184
OR (95%CI); *P*-value	1.0 (reference)	1.26(0.76,2.07); 0.372	0.87(0.29,2.62); 0.809	1.20(0.74,1.94); 0.464
TNM classification				
III+IV/I+II	151/154	105/83	9/23	114/106
OR (95%CI); *P*-value	1.0 (reference)	1.29(0.90,1.86); 0.171	**0.40(0.18,0.89); 0.021**	1.10(0.78,1.55); 0.601
Adenocarcinoma				
Yes/No	176/130	109/79	16/16	125/95
OR (95%CI); *P*-value	1.0 (reference)	1.02(0.71,1.47); 0.920	0.74(0.36,1.53); 0.414	0.97(0.69,1.38); 0.873
Rectal cancer				
Yes/No	239/135	129/59	14/18	143/77
OR (95%CI); *P*-value	1.0 (reference)	1.23(0.85,1.79); 0.267	**0.44(0.21,0.91); 0.024**	1.05(0.74,1.49); 0.788
**rs1143634**	CC	CT	TT	CT+TT
Tumor size				
≥5cm /<5cm	159/240	58/61	4/4	62/65
OR (95%CI); *P*-value	1.0 (reference)	1.44(0.95,2.17); 0.085	1.51(0.37,6.12); 0.562	1.44(0.96,2.15); 0.074
CEA				
Positive/ Negative	103/296	37/82	2/6	39/88
OR (95%CI); *P*-value	1.0 (reference)	1.30(0.83,2.03); 0.255	0.96(0.19,4.82); 0.958	1.27(0.82,1.98); 0.279
CA199				
Positive/ Negative	120/279	30/89	1/7	31/96
OR (95%CI); *P*-value	1.0 (reference)	0.78(0.49,1.25); 0.304	0.33(0.04,2.73); 0.282	0.75(0.48,1.19); 0.219
Differentiation				
Poor/Well+moderate	55/344	21/98	3/5	24/103
OR (95%CI); *P*-value	1.0 (reference)	1.34(0.77,2.32); 0.296	3.75(0.87,16.1); 0.057	1.46(0.86,2.47); 0.160
TNM classification				
III+IV/I+II	200/199	58/61	6/2	64/63
OR (95%CI); *P*-value	1.0 (reference)	0.95(0.63,1.43); 0.791	2.99(0.60,14.97); 0.164	1.01(0.68,1.51); 0.958
Adenocarcinoma				
Yes/No	243/156	55/64	2/6	57/70
OR (95%CI); *P*-value	1.0 (reference)	**0.55(0.37,0.83); 0.004**	0.21(0.04,1.07); 0.063	**0.52(0.35,0.78); <0.001**
Rectal cancer				
Yes/No	287/112	87/32	7/1	94/33
OR (95%CI); *P*-value	1.0 (reference)	1.06(0.67-1.68); 0.801	2.73(0.33-22.46); 0.330	1.11(0.71-1.75); 0.647

Bold values are statistically significant (*P* <0.05)

Finally, a prediction model of CRC was built using GRS and used to evaluate the effects of GRS on the CRC risk in a Chinese population (Table 5). Weighted GRS indicated the risk of CRC decreased in the tertiles quartile compared with the first quartile.

**Table 5:** The association of genetic risk score of IL-1B with risk of colorectal cancer.

Variable	Case (n = 527)		Control (n = 639)		OR (95% CI)		*P* value		Adjusted OR (95% CI)*		*P* value	
	N (%)	N (%)									
Weighted GRS											
First (0)	227 (43.1%)	227 (35.5%)		1				1			
Second (0)	247 (46.9%)	307 (51.2%)		0.81(0.63, 1.03)		0.087		0.81(0.63, 1.03)		0.090	
Third (0-0.5)	51 (9.7%)	101 (15.8%)		**0.51(0.34, 0.74)**		<0.001		**0.51(0.34, 0.74)**		<0.001	

The genotyping for rs1143623 and rs1143634 was successful in: 526 cases and 637 controls. *Adjusted for sex and age. Bold values are statistically significant (*P* < 0.05).

### Survival analysis of CRC patients with IL-1B gene rs1143634 or rs1143623 polymorphism

**Figure 3:**
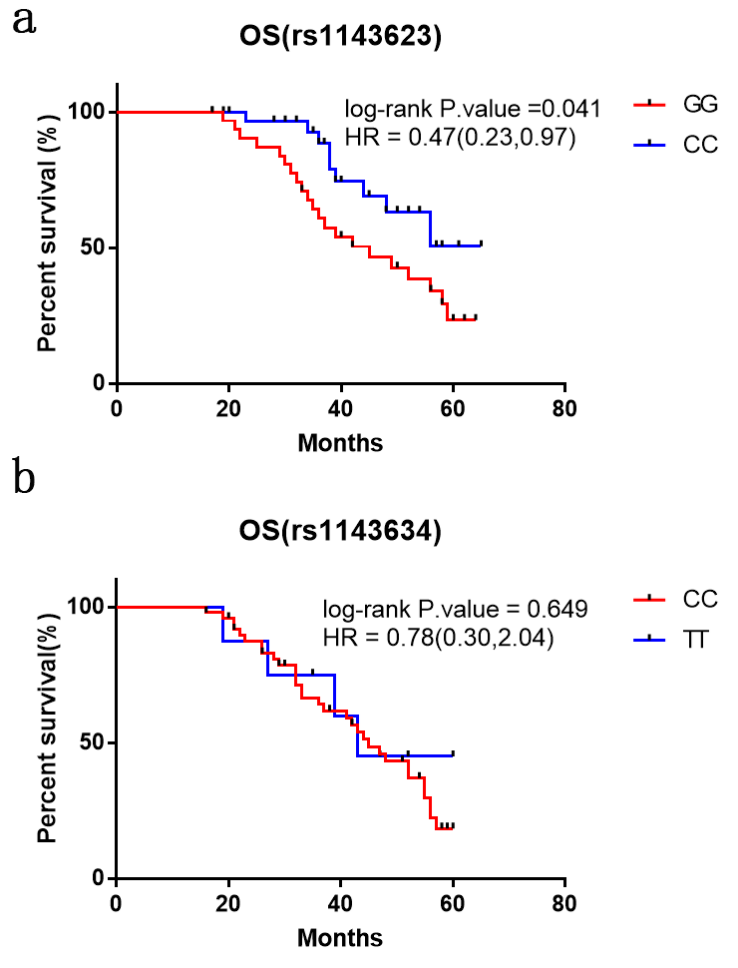
**Kaplan–Meier analysis of overall survival of CRC patients with IL-1B rs1143623 and rs1143634 polymorphisms.** Differences in the overall survival of the patients with different genotypes of polymorphisms (**a**) rs1143623 and (**b**) rs1143634.

Considering the protective roles of the two polymorphisms in CRC patients, we aimed to explore the relationship between SNPs and the prognosis of CRC patients. The 96 CRC patients enrolled in the first year of this study were followed up for a median period of 39 months. For rs1143623 polymorphism, the Kaplan-Meier single factor analysis showed that CRC patients carrying CC genotype versus GG genotype enjoyed longer OS (HR, 0.47, 95%CI, 0.23-0.97; log-rank *P* = 0.041, Figure 3). However, no difference in OS was found between the CRC patients carrying IL-1B rs1143634 TT and CC genotypes (HR, 0.78, 95%CI, 0.30-2.04; log-rank *P* = 0.649, Figure 3).

## DISCUSSION

The two IL-1B gene variants (rs1143634, rs1143623 polymorphisms) were found to decrease risk of CRC. Stratified analyses of age, sex, smoking, and drinking also showed positive findings. The rs1143623 polymorphism was associated with a larger tumor size, and TNM III+IV stages in CRC patients. Furthermore, the CC genotype of IL-1B gene rs1143623 polymorphism was related to better OS of CRC.

Many studies explored the association between IL-1B gene polymorphisms and CRC risk, but report conflicting findings. A prospective Danish case-cohort showed IL-1B gene rs1143623 polymorphism was associated with risk of CRC [10], but found no interaction between this SNP and diet or lifestyle factors [10]. A study from Russia observed similar findings in rectal cancer [11], and provided rs1143623 polymorphism was implicated in rectal cancer development, but not in CRC, gastric cancer, or ovarian cancer [11]. However, no association between rs1143634 polymorphism and CRC risk was found in a Caucasian population [12]. Burada et al. also failed to replicate significant finding in Romania [13], and found no positive results in the tumor site subgroup of CRC [13]. However, IL-1B gene rs1143634 polymorphism was associated with increased risk for CRC in a Chinese Han population [14]. Inconsistent with the above studies, our study showed both rs1143634 and rs1143623 polymorphisms of IL-1B gene provided protective roles in CRC development. We thought the conflict among the above studies may be attributed to the following factors. Firstly, genetic heterogeneity for CRC existed among races. Secondly, clinical heterogeneity was a potential factor. For example, the study from Russia only obtained significant findings in rectal cancer [11], while our study observed positive association in CRC, and the tumor sites were different between two studies. Thirdly, the sample sizes varied among these studies. Fourthly, diverse lifestyles and diets may also contribute to it. Fifthly, the differences of inclusion and exclusion criteria may contribute to the above conflict findings. The 244 CRC patients from Russia were verified by two experienced pathologists [11], but the study from Romania [13] involved standard diagnostic procedures including physical, digital rectal examination, fecal occult blood tests, sigmoidoscopy, colonoscopy, bariumenema, and imaging. The cases and controls from Denmark selected from The Diet, Cancer and Health Study did not mention the inclusion or exclusion criteria [10]. The study from China [14] indicated all CRC patients were diagnosed and histologically identified. To our knowledge, we are the first from China to uncover the protective effects of IL-1B gene rs1143623 and rs1143634 polymorphisms on CRC development.

Our stratified analyses suggested IL-1B gene rs1143623 and rs1143634 polymorphisms decreased the risk of CRC among females, smokers and drinkers, indicating these groups were more prone to the risk factors. However, these results should be interpreted with caution given the small sample size. Furthermore, we lso revealed the significant association between CC and/or GC genotype of rs1143623 polymorphism and clinical characteristics of CRC (tumor size ≥5cm, TNM III+IV stage and rectal cancer). No significant association between rs1143623 polymorphism and colon cancer was found, suggesting this SNP may be a specific locus for rectal cancer. Last but not least, the rs1143623 polymorphism was related to longer OS of CRC patients. Our data suggest rs1143623 and rs1143634 polymorphisms play protective roles in the pathogenesis of CRC but the underlying mechanisms are unclear. Bioinformatics analysis of the TCGA database showed the IL-1B gene mRNA levels were significantly increased in CRC patients compared with the controls. In addition, data from the International HapMap Project and GTEx portal showed IL-1B rs1143623 and rs1143634 polymorphisms could decrease the IL-1B expression. It is reasonable to assume that IL-1B rs1143623 and rs1143634 polymorphisms may contribute to the risk of CRC by altering the IL-1B expression.

This study has several limitations. Firstly, the sample size was not large enough, especially for the subgroup analyses. Secondly, the pathogenesis of CRC cannot be explained by only two SNPs in IL-1B gene and should be confirmed by other SNPs and gene variants. Thirdly, possible gene-gene or gene-environment interactions were not investigated. Fourthly, the findings may be inapplicable to other races. Fifthly, the association between these two SNPs and IL-1B protein should be explored. Sixthly, we did not follow up all patients due to the staff shortage.

In conclusion, IL-1B gene rs1143623 and rs1143634 polymorphisms are associated with decreased risk for CRC. Further studies in other ethnicities are needed to validate this finding.

## MATERIALS AND METHODS

### Study subjects

A total of 527 CRC patients were recruited at the Jiangsu Provincial of Traditional Chinese Medicine and the Third Affiliated Hospital of Nantong University. The diagnosis of CRC was confirmed by histopathological examination of the biopsy. The individuals with familial polyposis, inflammatory bowel disease or metastatic carcinoma were excluded. CRC patients were staged according to the American Joint Committee on Cancer/International Union against Cancer tumor-node-metastasis (TNM) staging system. The 639 healthy controls were selected from the volunteers undergoing general health check-up in the above two hospitals. The medical and follow-up data of patients, including age, gender, lymph node metastases, TNM stage, and location and differentiation degree of primary tumor were retrieved from hospital records and interviews. As for lifestyle factors, alcohol drinker was defined as an individual consuming alcohol more than once per day for ≥3 months, while smoker was defined as smoking at least once a day for ≥1 year.

This study was approved by the institutional review broads of the two hospitals and conducted in accordance with the Helsinki declaration. Written informed consent was obtained from all participants.

### Bioinformatic analysis

Data of IL-1B expression in CRC patients and healthy controls was extracted from GEPIA (http://gepia.cancer-pku.cn/index.html) [15], which is based on public data available from TCGA and is a web server for cancer and normal gene expression profiling and interactive analyses.

Genotype and mRNA expression data of the two IL-1B gene polymorphisms were cited from the International HapMap Project and GTEx portal (https://www.gtexportal.org/home/) [16], respectively.

### DNA extraction and Genotyping

In brief, 2mL of peripheral blood was collected in vacuum tubes containing 5% EDTA and frozen at -80 °C until use. Genomic DNA was extracted using a DNA Purification Kit (Tiangen Biotech) according to the manufacturer’s instructions. and the concentration and purity were estimated using NanoDrop at two optical density (OD) wavelengths 260 and 280 nm. Genotyping was done by matrix-assisted laser desorption/ionization time-of-flight mass spectrometry (MALDI-TOFMS) using a MassARRAY system (Sequenom, San Diego, CA, USA). Completed genotyping reactions were spotted onto a 384-well spectroCHIP (Sequenom) using a MassARRAY nano dispenser (Sequenom) and analyzed by MALDI-TOFMS. Genotype were called in real time on MassARRAY RT 3.1 (Sequenom) and analyzed on MassARRAY Typer 4.0 (Sequenom). For quality control, 10% of randomly-selected samples were analyzed repeatedly.

### Statistical analysis

All statistical analyses were performed on the SAS package (var. 9.1.3; SAS Institute, Cary, NC, USA). Deviation from the Hardy-Weinberg equilibrium (HWE) between observed and expected frequencies among controls was investigated using goodness-of-fit Chi-square test. Differences in demographic characteristics and lifestyle-related factors between cases and controls were compared by Chi-square test (for categorical variables) and Student’s t-test (for continuous variables). The impacts of different genotypes on other parameters were further evaluated via one-way analysis of variance (ANOVA). The odds ratio (OR) and 95% confidence interval (CI) were calculated to evaluate the SNPs-associated disease risk by binary logistic analysis. The effect size of each SNP was estimated using crude ORs. The weighted genetic risk scores (GRS) was equivalent to the sum of (log OR of SNP) × (number of risk alleles carried by the individual) across two SNPs. The GRS was subsequently divided into tertiles based on the normalized value. The association between GRS and CRC risk was then calculated by binary logistic regression. Univariate survival analyses were conducted via the Kaplan-Meier method. The overall survival (OS) was defined as date from diagnosis (colonoscopy or surgery) to death or the date last known alive. *P* ≤ 0.05 was considered statistically significant [17].
